# An Association Between Montessori Education in Childhood and Adult Wellbeing

**DOI:** 10.3389/fpsyg.2021.721943

**Published:** 2021-11-25

**Authors:** Angeline S. Lillard, M. Joseph Meyer, Dermina Vasc, Eren Fukuda

**Affiliations:** Department of Psychology, University of Virginia, Charlottesville, VA, United States

**Keywords:** wellbeing, human development, education, Montessori, positive psychology

## Abstract

Wellbeing, or how people think and feel about their lives, predicts important life outcomes from happiness to health to longevity. Montessori pedagogy has features that enhance wellbeing contemporaneously and predictively, including self-determination, meaningful activities, and social stability. Here, 1905 adults, ages 18–81 (*M* = 36), filled out a large set of wellbeing scales followed by demographic information including type of school attended each year from 2 to 17. About half the sample had only attended conventional schools and the rest had attended Montessori for between 2 and 16 years (*M* = 8 years). To reduce the variable set, we first developed a measurement model of wellbeing using the survey data with exploratory then confirmatory factor analyses, arriving at four factors: general wellbeing, engagement, social trust, and self-confidence. A structural equation model that accounted for age, gender, race, childhood SES, and years in private school revealed that attending Montessori for at least two childhood years was associated with significantly higher adult wellbeing on all four factors. A second analysis found that the difference in wellbeing between Montessori and conventional schools existed even among the subsample that had exclusively attended private schools. A third analysis found that the more years one attended Montessori, the higher one’s wellbeing as an adult. Unmeasured selection effects could explain the results, in which case research should determine what third variable associated with Montessori schooling causes adult wellbeing. Several other limitations to the study are also discussed. Although some of these limitations need to be addressed, coupled with other research, including studies in which children were randomly assigned to Montessori schools, this study suggests that attending Montessori as a child might plausibly cause higher adult wellbeing.

## Introduction

Wellbeing, or the felt experience of health, happiness, and flourishing, predicts several desirable outcomes including better health and work performance, longevity, and more positive social behavior and relations (Ryff, 2014). Low levels of wellbeing predict suicidal behavior even more strongly than does mental illness (Keyes et al., 2012). Even intrinsically, wellbeing could be considered the supreme human outcome (Diener et al., 2015). Although wellbeing is partially determined by genetic inheritance (Røysamb and Nes, 2019), environmental factors are important contributors (Diener et al., 2016). Yet few childhood experiences have been shown to predict adults’ psychological wellbeing. One that does is residential moves: more moves in childhood significantly predicts lower adult wellbeing (Oishi and Schimmack, 2010). Here we explore whether a different childhood experience, Montessori education, might predict higher adult wellbeing. We know of no research examining an association between Montessori specifically and later wellbeing, but one study found that people who had attended various alternative schools including Montessori as children adjusted better to university, controlling for high school baseline (Shankland et al., 2010). Montessori warrants further study, as it is the most common and long-lasting alternative progressive pedagogy in the world (Debs, 2019) and has several features that are endemic to wellbeing-enhancing educational environments (White and Kern, 2018).

A logic model for Montessori education (Culclasure et al., 2019) predicts that Montessori features like choosing one’s activities, using real, hands-on materials, and collaborating with peers would result in a range of positive personal and social outcomes. Summaries of child development research and their implications for educational environments also suggest that attending schools with Montessori features (like collaboration and learning based on interests) should enhance wellbeing (National Academies of Sciences Engineering and Medicine, 2018; Darling-Hammond et al., 2019). Actual studies in conventional schools also show that features consistent with Montessori (like low test anxiety: Montessori has no tests) predict higher wellbeing in school (Baker, 2004; Cohen, 2006; Felner et al., 2007; Seligman et al., 2009; Steinmayr et al., 2016, 2018). Furthermore, random lottery studies of Montessori students (discussed later) show differences from waitlisted controls suggesting Montessori lays groundwork that would be expected to lead to higher wellbeing (Lillard and Else-Quest, 2006; Lillard et al., 2017). Here we present a series of structural equation models showing that Montessori schooling in childhood is associated with higher adult wellbeing, after accounting for a range of demographic variables. We begin with discussion of the Montessori system and three features that are known to enhance wellbeing in school and other settings: choice or self-determination, meaningful activities, and social stability.

### Montessori Schooling

**FIGURE 1:**
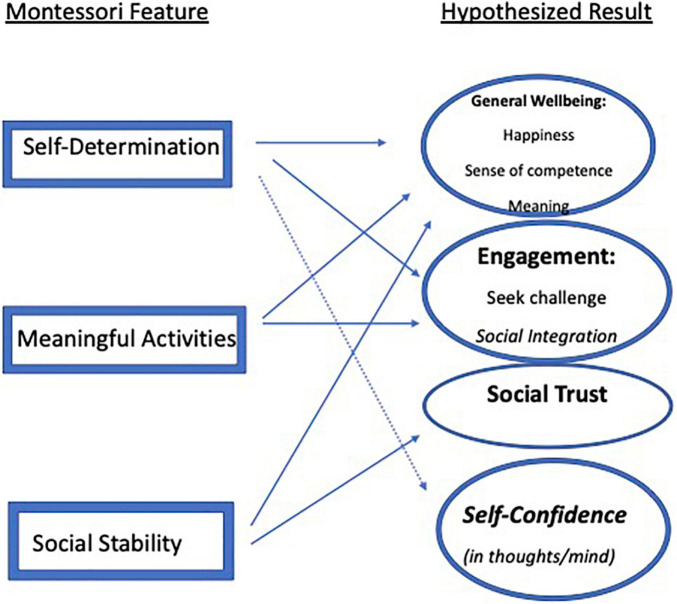
Final model of hypothesized relation of Montessori features to wellbeing aspects. This is the final model that was arrived at after analyses; the hypothesized model described in the text has a different set of hypothesized results.

Initiated in 1907, Montessori pedagogy (Montessori, 1967/1995) is the oldest surviving and most prevalent child-centered, constructivist education system in the world (Debs, 2019), practiced in over 500 public and thousands of private American schools (National Center for Montessori in the Public Sector, 2014) and tens of thousands of schools around the world (180 Studio and Saunders Eckenhoff, 2020). Although some think of it as a preschool model serving mainly White children, Montessori extends through high school, and over half of American public Montessori students today are children of color (Debs, 2019). Three salient Montessori features would be expected, based both on logic and prior research, to lead to certain long term wellbeing outcomes: self-determination, meaningful activity, and stable social relationships (for more discussion of characteristics of Montessori programs, see Lillard and McHugh, 2019a,b). Although we could not study this directly, we could examine whether there are associations between prior Montessori schooling and adult wellbeing. No study to our knowledge has specifically examined whether attending Montessori is associated with feelings of wellbeing over the long term; school studies tend to look at concurrent or relatively proximate outcomes (Baker, 2004; Cohen, 2006; Felner et al., 2007; Seligman et al., 2009; Steinmayr et al., 2016, 2018), or other long-term outcomes like income (Chetty et al., 2018). Wellbeing in adulthood is a multidetermined outcome, influenced by health, wealth, marital status, and many other features (Diener et al., 2013, 2016), but childhood school experience could plausibly be another predictor. Here we consider how Montessori embodies three features that other research has shown predict wellbeing. Figure 1 portrays the model we arrived at after conducting the study; below we describe the model we hypothesized prior to analyzing our data.

#### Self-Determination

Children in Montessori classrooms choose their own work almost all the time, and in this sense are very much in charge of their own educations. A teacher guides children in individual or small group lessons, but children decide which lessons to follow up on, and even whether to sit in on a lesson: they determine their own activities. The classroom is composed of hundreds of potential activities, laid out on shelves, and children choose among them, or even arrange their own field trips during which they leave the classroom to do something else (Montessori, 1967/1995; Lillard, 2017).

Self-determination has been conceptualized as fulfilling a fundamental human need for autonomy (Deci and Ryan, 2011). Choice and its progeny, a sense of control or agency, have been shown to predict stronger intrinsic motivation, self-efficacy, happiness, and sense of competence (Langer and Rodin, 1976; Ryan and Grolnick, 1986; Rovee-Collier and Hayne, 2000; Patall et al., 2008, 2010); living in a more individualistic country, where self-determination is higher, also predicts wellbeing at a national level (Rhoads et al., 2021). Related to self-determination, Montessori has no grades or tests. Research has shown that external rewards and performance evaluations proffer an external sense of control (Ryan and Deci, 2000), and their absence thus allows for an internal locus of control and a sense of self-determination (see Lillard, 2017, Chapter 6). In addition, when given external rewards for doing tasks, people tend to opt for easier tasks (Harter, 1978), avoiding challenge.

We hypothesized that self-determination might lead to two sets of outcomes. A first set concerns what one might call general wellbeing: happiness, finding meaning in life, self-confidence, and (relatedly) a sense of personal competence. A second set of potential outcomes is related to intrinsic motivation: people who experience a high degree of self-determination early in life might later be more intrinsically engaged and seek more challenge. Supporting the idea that self-determination in Montessori schools might cause this array of outcomes, an experience sampling study showed that Montessori middle school students rated themselves more highly than did conventionally schooled students on positive affect, experiences of “flow” (Csikszentmihalyi, 1997), potency, and intrinsic motivation (Rathunde and Csikszentmihalyi, 2005a). We hypothesized that adults who formerly attended Montessori schools would show higher general wellbeing, including self-confidence, and be more apt to seek challenge and be engaged with their work.

#### Meaningful Activities

Montessori offers children meaningful activities, by which we mean activities for which the underlying reasons are clear, and thus give people a sense of purpose. Offering meaningful activities is crucial for a school system built on self-determination (Lillard, 2019), because meaning motivates engagement (Bruner, 1990). In prekindergarten, Montessori children learn to prepare, serve, and clean up meals, take care of their classroom, and button their clothes—all meaningful activities for a young child; research shows that children prefer really doing such activities to pretending to do them, because they like having a purpose in their activities (Taggart et al., 2018, 2020). When learning abstract concepts like number, children in Montessori use concrete materials that make obvious what the abstraction is about; this extends for example to using a cube composed of 27 blocks that make clear why the trinomial formula gives the area of a cube: each block represents an element of the formula. If I see that putting together a set of blocks where three blocks have all sides of length A, B, and C (respectively), 3 correspond to a2b, and so on, then I can understand why the trinomial formula works; the formula then has meaning—it is not merely an abstraction. In addition, Montessori students can pursue activities that interest them, which of course translates to their activities being personally meaningful.

Work that is geared at an optimal level—challenging but possible—engages people, putting them in the positive state referred to as “flow” (Csikszentmihalyi, 1997). Under free conditions in which a range of meaningful options are supplied, and no rewards (like grades) are offered, people tend to choose engagements at an intermediate level of challenge (Danner and Lonky, 1981; Dweck, 1999; Simon, 2001; Kidd et al., 2012, 2014)—not so easy that they learn nothing, but not so difficult that they will collapse in frustration (and therefore not learn). Given this, the fact that children choose their own work should translate to work being meaningful, which would in theory support higher levels of engagement in Montessori schools. The aforementioned research also suggests this is the case (Rathunde and Csikszentmihalyi, 2005a): Montessori middle school students reported feeling more engaged than other students. Engagement also increases general wellbeing (Lewis et al., 2011), and so by extension meaningful activities might also increase general wellbeing. As shown in Figure 1, we hypothesized that the increased engagement that research has shown occurs in Montessori classrooms, perhaps due to one’s activities being meaningful, translates to increased engagement in activities throughout life. We also expected that a pattern of having meaningful activities in the school years could translate to a general sense of meaning in life and happiness, and thus be related to general wellbeing.

#### Social Stability and Cohesion

Another Montessori education feature that might enhance long term wellbeing is the social environment, which nourishes social-emotional development and sustained relationships. Classrooms span 3 years (for example, 6- to 9-year-olds, 9- to 12-year-olds, and so on through high school) during which children have the same teacher and immediate peer group; children just older and younger are classmates for 1–2 years, and are met again as one moves up classroom levels.

The practice of staying with the same teacher and classmates, called “looping,” supports positive relationships, self-confidence, and academic performance (Burke, 1996; Little and Dacus, 1999; Cistone and Shneyderman, 2004; Nitecki, 2017; Hill and Jones, 2018); the one study that showed better academic performance but not better relationships (Tourigny et al., 2020) used only 2 years of looping whereas Montessori and some other studies use 3; 3 years (versus 2) might make a difference to relationship quality. Positive academic performance also predicts later wellbeing (Ng et al., 2015; Steinmayr et al., 2016). Being in ungraded multiage classrooms (for example, where “1st grader” is not identified), as Montessori children are, is also particularly beneficial for both academic and social-emotional outcomes, and the benefits increase with more time in such classrooms (Lloyd, 1999).

The Montessori practice of not having grades or tests also might benefit relationships with both teachers and peers. For one, the teacher becomes a guide, rather than one who makes judgments and “grades.” Among students, absence of tests and grades fosters collaboration whereas grades foster competition (Butler and Ruzany, 1993). Collaboration itself is another reason why Montessori schooling might be associated with social aspects of higher adult wellbeing. In Montessori classrooms, particularly at after age 6, students constantly collaborate on schoolwork, which could reasonably be expected to cultivate social skills. As noted, older Montessori students also go on self-arranged small group field trips, and they often create classroom rules themselves (as a group). By middle and high school, Montessori classrooms might go on longer trips together. All these practices could foster greater social cohesion.

We know of no strong studies examining child-teacher relationships in Montessori, but there is evidence (including from lottery-control studies, discussed below) suggesting that peer relationships are stronger in Montessori. This makes sense because, as opposed to conventional schools where students usually work individually, in Montessori schools students often work in small groups. Moreover, research indicates that Montessori student’s social knowledge and skills are more advanced, and the overall school climate is better (Flynn, 1991; Rathunde and Csikszentmihalyi, 2005b; Lillard and Else-Quest, 2006; Lillard et al., 2017; Denervaud et al., 2020a). Random lottery studies also indicate that academic performance is stronger in Montessori (Lillard and Else-Quest, 2006; Lillard et al., 2017) and well-controlled matched/growth studies (e.g., Culclasure et al., 2018; Denervaud et al., 2019) suggest Montessori leads to higher academic performance. Stronger academic performance has been shown to lead to higher well-being (Ng et al., 2015; Steinmayr et al., 2016), possibly *via* improved self-esteem (Yang et al., 2019), which reinforces more positive relationships and sense of community.

Taken together, findings on social stability led us to predict that Montessori students would have more positive social relationships and a stronger sense of community throughout life (see Figure 1). Such factors are typically related to general wellbeing, thus the strong social stability in Montessori schools could also predict higher general wellbeing later.

#### Summary

In sum, we hypothesized that Montessori pedagogy in childhood might lead to higher wellbeing later in life. The reasoning behind this hypothesis was that the pedagogy has three features (self-determination, meaningful activities, and social cohesion) that other studies have shown enhance wellbeing along several dimensions (clustering into what we might call general wellbeing, intrinsic motivation/engagement, and social skills/social cohesion), and because studies of Montessori versus conventionally schooled children (including natural experiments) have indicated that Montessori students are different along these dimensions during their school years. In the next section we present those natural experiments.

### Natural Experiments Suggesting Montessori Might Cause a Trajectory to Higher Wellbeing

Natural experiments using random lotteries have examined the immediate influence of Montessori on proximal child outcomes, and the results for the two studies which involved highly trained Montessori teachers suggest children have higher wellbeing while in school and are on a trajectory toward higher wellbeing later in their lives. These studies controlled for parent characteristics because admission was determined by a random lottery that parents of the control children had also entered. The studies thus have high internal validity, although the results might not apply to families that do not enter such lotteries, lowering external validity. The first study examined children in Kindergarten and 6th grade (Lillard and Else-Quest, 2006), whereas the second followed across preschool (from ages 3 to 6) children who were equal on all measured outcomes at baseline (Lillard et al., 2017). Both contrasted children in high fidelity public Montessori schools (in that both met the standards for recognition by the Association Montessori Internationale or AMI, which meant all the teachers had AMI’s intensive 9-month training and diploma) with waitlist control children in business-as-usual non-Montessori schools. In terms of self-determination and its benefits, the studies showed better academic performance and mastery orientation. In terms of social skills, they showed better social cognition and behavior, and a stronger sense of community. They also indicated more developed executive function. Academic performance, mastery orientation, social skills, and executive function all predict higher wellbeing (Moffitt et al., 2011; Reynolds et al., 2011, 2017; Sancassiani et al., 2015; Steinmayr et al., 2016, 2018; Haimovitz and Dweck, 2017; Darling-Hammond et al., 2019). Because random assignment designs support causal inference, these lottery control studies suggest attending Montessori might cause higher wellbeing in adults. However, a third natural experiment in France contrasted Montessori children taught by untrained teachers with Ecole Maternale (the highly regarded national preschool program) children; all were randomly assigned at the classroom level (Courtier, 2020). In this study, Montessori children performed unequivocally better on reading, but not on an array of other measures. Further research is needed to determine why these studies had different results; one possibility is that wellbeing-related qualities emerge more reliably when teachers have Montessori training; another is that the Ecole Maternale program has superior outcomes to business-as-usual programs in the United States. Regardless, the U.S. studies lend support to the idea that Montessori causes certain qualities in American children, and other studies show those qualities to be associated with higher wellbeing.

Taking together these findings, as well as the fact that Montessori has conditions that are associated with higher wellbeing, we hypothesized that adults who went to Montessori as children have higher adult wellbeing.

## Materials and Methods

### Participants

Participants were recruited through various methods including Facebook ads in cities known to have many Montessori schools, Amazon’s Mechanical Turk, school associations, and snowballing. The final sample consisted of 1905 participants in the US and Canada who had attended Montessori for at least 2 years or who had spent virtually all their school years at conventional schools. Two years of intervention was selected because that duration led to significant outcomes for the Perry Preschool Project (Heckman, 2006), and 2 years of Head Start is significantly more impactful than 1 year (Wen et al., 2012). Although Montessori schooling considers 3 years in a classroom to constitute a full “cycle,” whether specific sets of 3 years are associated with later wellbeing was not addressed here. Participants’ mean age was 37.05 years (*SD* = 13.12, range = 18–81 years), 79.2% were female, and 83.0% identified as White, 3.4% as Black or African American, 4.5% as Asian, 3.7% as Hispanic or Latino, and less than 1% as American Indian or Alaska Native, Native Hawaiian or Pacific Islander. Another 3.8% aligned with multiple races/ethnicities, and 1% self-identified in other categories (e.g., Jewish) or preferred not to answer. Because of the small sizes of categories other than White, they were grouped for analyses.

The sample size goal was 2000 participants, 1000 in each group (Montessori and conventional), which is well above the threshold needed for an SEM involving 83 paths which is the size of our largest model (Wolf et al., 2013). Our recruiting cut-off was set at the desired *n*s or 6 months, whichever came earlier. Based on Wolf et al.’s (2013) analysis, and given our model structure, parameters, and variables, our final sample size of 1905 should have sufficient power to detect the hypothesized effects.

Participants were partitioned into groups using R. Those who had spent no or only 1 year in an unconventional school (like Montessori) were categorized as conventional, *n* = 1071 (19 had gone to Montessori for just 1 year). Those with at least 2 years of Montessori, *n* = 834, were classified as such. Although a 2-year cutoff was used, for the Montessori group the average length of attendance was 8 years (*SD* = 3.66, range 2–16). An additional group of participants who had attended other alternative schools for two or more years (*n* = 506) were excluded from analyses to focus here on Montessori versus conventional schooling. (Their results will be reported elsewhere).

### Procedure

Surveys were administered on the Qualtrics platform with a compensation rate of $0.50/survey. Participants who gave informed consent were then given a series of scales and questions, ending with demographic questions including school types attended. The stated purpose of the research in the informed consent was “to better understand the long-term outcomes of alternative and conventional school education on peoples’ lives.” On the final page, school type history was requested; no specific school system was mentioned until after all other measures had been filled out.

#### Survey

The survey included 18 established scales (see below) that were intended to get at a variety of aspects of wellbeing, and a few ordinal scales and other questions getting at issues of interest. Because the ordinal scales explained little variance in wellbeing, they were not used in our analyses, but those results were consistent with the ones below and will be reported elsewhere. Eleven of our 18 scales are subscales of the Psychological and Social Wellbeing scales (SWB). Below, after considering advantages and disadvantages of online surveys, we describe each scale with its original alpha; in the present study, alphas were the same or exceeded the originals in all cases except one (Social Coherence, for which our α = 0.54 vs. 0.64 in the original).

#### Online Survey Delivery

Online surveys are an important source of data for psychology research, with pros and cons (Gosling and Mason, 2015). One benefit is the ability to recruit large samples from the general population (versus, for example, undergraduate psychology majors) including samples with relatively rare characteristics of interest. This made them ideal for this study, since Montessori schools are much less common than conventional schools. Other benefits are reducing self-presentation bias and experimenter influence. There are also disadvantages. For example, drop-out rates in online survey research are high, averaging 34% in a meta-analysis that saw ranges from 0 to 87% (Musch and Reips, 2000). Drop-out can be a serious concern when it occurs more in one assigned condition than another, but this does not apply here because conditions were not assigned in this study. Also mitigating this concern, studies have shown that samples completing internet surveys closely resemble the populations from which they are drawn (Gosling and Mason, 2015). Another potential problem is multiple submissions; we guarded against this by ensuring each respondent had a unique computer identifier (IP address). Another issue is that respondents are limited to people who use the internet, and findings might not generalize to the population. As internet usage increases, this is less of a concern—87% of households in the developed world were on the internet in 2018 and these data were collected in 2019 (ITU Publications, 2019). However, it is the case that survey respondents (be they on a telephone or online) tend to be younger, female, White, and affluent (Curtin et al., 2000; Singer et al., 2000; Smith, 2008). We accounted for these factors in our models, but it is a limitation of the online survey method and therefore of this study.

There are many measures of wellbeing (Ackerman et al., 2018), tapping into outcomes ranging from finding meaning in one’s life to mindful awareness to one’s sense of community support. Our approach was to use a wide range of accepted measures of adult wellbeing, and reduce the measures to a manageable set using exploratory and confirmatory factor analyses, and then examine how the resulting factors align with the three hypothesized outcome clusters. Finally, we conducted a series of Structural Equation Modeling (SEM) analyses to examine whether Montessori was a meaningful predictor of outcomes after accounting for demographic variables.

#### Psychological Wellbeing Scales (PWB)

Participants filled out six PWB scales (Ryff and Keyes, 1995) of three items each. Participants rated each item using a 7-point scale ranging from *strongly agree* to *strongly disagree*. The six PWB scales, with their original reported alphas, are Self-Acceptance (e.g., “When I look at the story of my life, I am pleased with how things have turned out so far”; α = 0.59), Environmental Mastery (“I am good at managing the responsibilities of daily life”; α = 0.52), and Autonomy (“I judge myself by what I think is important”; α = 0.48), all of which seem to tap into General Wellbeing (see Figure 1); Personal Growth (“Life is a continuous process of growth”; α = 0.55) and Purpose in Life (“Some people wander aimlessly through life; I am not one of them”; α = 0.36), which seem to tap Engagement; and Positive Relations (“People would describe me as a giving person”; α = 0.58), which seems to tap Social Trust. Higher scores indicate greater levels of wellbeing.

#### Social Wellbeing Scales

The five SWB Scales (Keyes, 1998) each have three items that use the same 7-point scale as the PWB scales. The SWB scales are Social Coherence (“I can predict/make sense of the world”; α = 0.64) and Social Contribution (“I have something to give”; α = 0.66) which tap into the self-confidence aspect of General Wellbeing and perhaps the meaning aspect of Engagement; and Social Integration (“I feel close to people in my community”; α = 0.73), Social Acceptance (“People are kind”; α = 0.41), and Social Actualization (“Society is getting better”; α = 0.64) which all appear to tap into Social Trust.

#### Satisfaction With Life Scale

The Satisfaction with Life Scale (SWL) (Diener et al., 1985), one of the most commonly used measures of wellbeing (Ackerman et al., 2018), consists of five items (e.g., “In most ways my life is close to my ideal”; α = 0.87) which participants rate using the same 7-point scale ranging from *strongly disagree* to *strongly agree*. Ratings for each of the five items are summed up to calculate a single aggregate score. A high score indicates high satisfaction with one’s own life, and seems to tap into one’s general sense of wellbeing and happiness.

#### Meaning in Life Questionnaire

This 10-item scale (Steger et al., 2008) measures meaning in life, including its presence and one’s search for meaning; in the current study we used the 5-item MILQ-Presence subscale to assess the presence of meaning in life. Using a 7-point scale ranging from *absolutely untrue* to *absolutely true*, participants rate five short statements such as, “My life has a clear sense of purpose”; α = 0.86. An aggregate score is calculated by summing up the five items, and a higher score reflects a higher subjective sense of meaning in one’s life. This scale appears to tap into the meaning aspect of engagement.

#### Subjective Vitality Scale

This 7-item scale (Ryan and Frederick, 1997) measures the extent to which one feels alive and alert. Using a 7-point scale ranging from *not at all true* to *very true*, participants rate seven short statements such as, “I feel alive and vital”; α = 0.83. An aggregate score is calculated by adding ratings from each of the items. As is recommended, the second item (the only one needing to be reverse scored) was dropped (Bostic et al., 2000). This scale also seems to tap general wellbeing and happiness.

#### Short Need for Cognition Scale

This 18-item scale (Cacioppo et al., 1984) measures the extent to which individuals engage in and enjoy effortful thinking. Using a 5-point scale ranging from *extremely uncharacteristic of me* to *extremely characteristic of me*, participants rate statements such as, “I really enjoy a task that involves coming up with new solutions and problems”; α = 0.90. An aggregate score is calculated by adding ratings from the 18 items, with higher scores indicating high interest in thinking, complex problem solving, and intellectual tasks. We expected that having the opportunity to choose difficult and meaningful activities as a child might lead to a lifelong desire to seek challenges, creating Engagement.

#### Mindful Attention Awareness Scale

This 15-item scale (Brown and Ryan, 2003) measures individuals’ dispositional mindfulness, or awareness and attention to the present moment. Using a 6-point scale ranging from *almost always* to *almost never*, participants rate each statement with reference to their day-to-day experiences. For example, one item is, “I find it difficult to stay focused on what is happening in the present;” a high score means that is almost never true; α = 0.81. An aggregate score is calculated by averaging the 15 responses. Higher scores reflect higher dispositional mindfulness, which is thought to be generally related to wellbeing.

As noted we also administered some ordinal scales and a few isolated questions; in addition we administered the Big 5 personality survey (Brody and Ehrlichman, 1998). Because whether personality should be viewed as a wellbeing *outcome* is controversial, we do not discuss this further here, nor do we discuss the ordinal scales which did not contribute to the variance in our models.

#### Demographics and School History

After completing the wellbeing measures, participants answered standard demographic questions, reporting factors such as their age, gender, and race. In addition, they were asked how they would describe their family’s social class when they were 3–12 years old, with five options: lower/working, lower middle, middle, upper middle, and upper. Similar scales have been successfully used in other studies to measure adults’ estimates of their childhood SES (Straughen et al., 2013; Listl et al., 2018; Lindberg et al., 2021). Childhood SES is highly related to child outcomes (Reardon, 2011; Duncan and Murnane, 2014), and a meta-analysis showed that one’s own estimate of one’s SES (often called SSS, for subjective social status) is more strongly related to wellbeing than is one’s actual SES (Tang et al., 2016).

Finally, participants were asked, for each year from ages 2 to 17, what type of school they attended, with options including Regular/Traditional, Montessori, Homeschool, Waldorf, Reggio Emilia, Other Alternative, and did not attend. They were also asked how the school they went to each year was funded, with options of Public, Private (non-religious), and Private (religious), and did not attend. The format of this page was as follows: the ages (“2 years old,” and so on) were listed down the left-most column, the next seven columns held circles one could check for the type of school, and the next four columns were for the funding model.

### Analytic Approach

There was no missing data. An exploratory factor analysis (EFA) was done on a randomly selected two-thirds of respondents’ data using all variables and maximum likelihood extraction. A four-factor structure was confirmed in a confirmatory factor analysis (CFA) done with the remaining data. These factors were then entered in a series of Structural Equation Models (SEM) that accounted for age, race, childhood SES, gender, and proportion of years attending private schools. All analyses were run using R, OpenMx 2.19.1 (Neale et al., 2016), and SPSS 24.

#### Model Fit

The purpose of using factor analysis here was to reduce the variable set; the variables were selected because they are frequently used to measure wellbeing, rather than with an eye to expected factors. Adequacy of model fit was determined according to the guidelines set by Plucker (2003) for factor analyses (RMSEA.05-0.10; AGFI 90+, CFI.90+) and using a multi-index approach as suggested by Hu and Bentler (1999). For this we added the Tucker-Lewis Index (TLI), adopting the common standard for of 0.90. RMSEA was the favored index because of the relatively large sample size and number of indicators, which can lower the values of CFI and TLI indices (Kenny and McCoach, 2003; Shi et al., 2019). Chi-square was inappropriate here because of the relatively large sample size (Hu and Bentler, 1999; Russell, 2002).

### Data Preparation

Several scales, particularly the PWB scales, resulted in data that were on visual inspection negatively skewed, reflecting that as a whole, the sample had high wellbeing. Box–Cox transformations were applied to skewed variables (Box and Cox, 1964). We rounded lambdas to the nearest whole number to decide whether to square or cube the variable. Box–Cox transformations were then applied to check that the new rounded lambda value was close to 1; the one exception to this was Personal Growth, which would need to be raised to the 6th power by this criterion; after cubing had a lambda of 2.07 and was not transformed further. As stated, there were no missing data.

## Results

Table 1A shows the raw unadjusted means, SDs, scale alphas in this study, and the correlations among the scales, age, and Montessori status; Table 1B shows the means and SDs or percentages on each demographic variable for the Montessori and conventional samples separately, and Table 1C shows the same for the scales. The vast majority (80%) of participants filled out the surveys in under 30 min; 16 min was the modal time to completion. First we report results from the EFA, then the CFA. We then discuss the resulting factors before turning to the SEM.

**TABLE 1A:** Means, SDs, and correlation matrix for observed continuous variables: entire sample.

Observed variable	Mean	*SD*	α	1	2	3	4	5	6	7	8	9	10	11	12	13	14	15	16	17	18
1. Age	37.05	13.11		1.0																	
2. Yes/no Montessori	–0.12	0.99		–.34	1.0																
3. Life satisfaction	24.95	7.10	0.90	0.03	0.16	1.0															
4. Self acceptance	16.82	3.90	0.75	0.05	0.18	0.75	1.0														
5. Meaning in life	26.64	6.55	0.91	0.19	0.02	0.59	0.60	1.0													
6. Environ. mastery	15.57	3.86	0.69	0.12	0.17	0.62	0.66	0.48	1.0												
7. Subjective vitality	4.71	1.26	0.90	0.10	0.13	0.60	0.59	0.55	0.59	1.0											
8. Autonomy	16.87	3.16	0.56	0.24	–0.03	0.20	0.30	0.27	0.33	0.20	1.0										
9. Mindful awareness	4.08	0.79	0.87	0.24	0.05	0.31	0.39	0.36	0.46	0.41	0.34	1.0									
10. Social coherence	13.57	3.77	0.54	0.03	0.14	0.30	0.35	0.29	0.40	0.31	0.30	0.29	1.0								
11. Personal growth	19.05	2.59	0.67	–0.08	0.23	0.39	0.48	0.42	0.44	0.45	0.23	0.25	0.24	1.0							
12. Purpose in life	17.05	3.24	0.42	–0.04	0.15	0.43	0.51	0.46	0.41	0.36	0.22	0.27	0.27	0.51	1.0						
13. Positive relations	17.01	3.80	0.65	0.07	0.20	0.51	0.57	0.46	0.48	0.46	0.22	0.35	0.27	0.43	0.41	1.0					
14. Social integration	16.48	4.49	0.87	0.03	0.25	0.50	0.54	0.45	0.45	0.47	0.13	0.25	0.24	0.45	0.38	0.59	1.0				
15. Social actualization	13.35	4.24	0.71	–0.13	0.24	0.38	0.38	0.25	0.32	0.36	0.05	0.18	0.35	0.32	0.26	0.32	0.38	1.0			
16. Social acceptance	14.23	3.55	0.51	0.04	0.22	0.37	0.36	0.27	0.33	0.32	0.12	0.19	0.23	0.33	0.22	0.39	0.45	0.51	1.0		
17. Social contribution	18.02	3.38	0.76	0.11	0.19	0.48	0.59	0.57	0.48	0.48	0.28	0.29	0.34	0.55	0.49	0.47	0.57	0.35	0.39	1.0	
18. Short need cognit.	69.14	12.03	0.90	0.03	0.12	0.26	0.28	0.26	0.27	0.31	0.30	0.20	0.32	0.43	0.32	0.22	0.24	0.20	0.21	0.40	1.0

**TABLE 1B:** Means, SDs, and/or percentages for demographic variables for the Montessori and conventional samples.

	Montessori	Conventional
*Mean Age (SD)*	31.95 (*9.6*)	41.0 (*14.1*)
*Gender* Male	23.3	19.0
Female	76.7	81.0
*Race/Ethnicity* White	83.8	82.4
Black or African American	1.8	4.6
American Indian or Alaska Native	0.2	0.7
Hispanic or Latino	3.7	3.7
Asian	4.1	4.9
Native Hawaiian or Pacific Islander	0.5	0.0
Multiple	5.4	2.6
Other	0.2	1.0
Preferred not to answer	0.2	0.1
*Maternal education*		
Less than high school diploma	1.4	7.0
High school diploma	5.0	24.0
Some college or vocational training	9.1	16.1
2-year college degree	4.4	9.1
4-year college degree	35.1	26.5
Post-college degree	44.8	17.4
*Social class during childhood*		
Lower/working	5.2	16.5
Lower middle	10.8	19.4
Middle	40.2	38.6
Upper middle	38.8	22.7
Upper	5.0	2.8
*Proportion private schooling*	0.67	0.37
*Years in Montessori*	8.03 *(3.7)*	0.02 *(0.13)*
*Years in conventional*	7.22 *(3.8)*	14.0 *(1.34)*

*Standard deviations (SDs) are in italics.*

**TABLE 1C:** Untransformed means and standard errors for Montessori and conventional samples.

	Montessori		Conventional	
	*M*	*(SE)*	*M*	*(SE)*
Psychological wellbeing				
*Personal growth*	19.73	*(0.07)*	18.52	*(0.09)*
*Environmental mastery*	16.31	*(0.12)*	14.99	*(0.12)*
*Purpose in life*	17.61	*(0.10)*	16.62	*(0.10)*
*Positive relations*	17.87	*(0.12)*	16.35	*(0.12)*
*Self-acceptance*	17.60	*(0.12)*	16.22	*(0.13)*
*Autonomy*	16.76	*(0.11)*	16.96	*(0.10)*
Social wellbeing				
*Social coherence*	14.18	*(0.12)*	13.09	*(0.12)*
*Social integration*	17.76	*(0.12)*	15.49	*(0.15)*
*Social acceptance*	15.12	*(0.11)*	13.54	*(0.11)*
*Social contribution*	18.73	*(0.09)*	17.47	*(0.11)*
*Social actualization*	14.50	*(0.13)*	12.44	*(0.13)*
*Satisfaction with life*	26.21	*(0.22)*	23.98	*(0.23)*
*Meaning in life*	26.78	*(0.21)*	26.53	*(0.21)*
*Subjective vitality*	4.90	*(0.04)*	4.57	*(0.04)*
*Mindful attention*	4.13	*(0.03)*	4.05	*(0.03)*
*Need for cognition*	70.75	*(0.37)*	67.90	*(0.40)*

*Scales used in factor analyses and standard errors are in italics.*

### Factor Analyses

Exploratory factor analysis using maximum likelihood extraction was run with the correlation matrix from data of approximately 2/3 of the participants (*n* = 1220). This *n* allowed covariates (such as gender and childhood SES) to be balanced across conditions (Montessori and conventional). A Promax oblique rotation was used, allowing all factors to intercorrelate. Initially the training set was run using 1–11 factors and all variables. The ordinal items (classified as “Other Questions” in the survey) explained little variance (low communality or *h*^2^ values), which made sense in that they were designed to get at issues a step removed from wellbeing. The ordinal variables were removed, and a new set of EFAs were run using the same methods.

**FIGURE 2:**
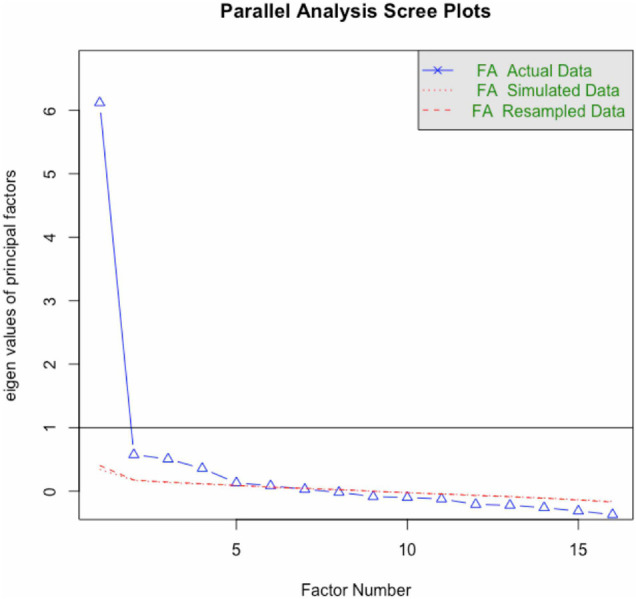
Parallel analysis scree plot.

A parallel analysis approach (Horn, 1965) was taken to determining the number of factors. This approach is favored over the Kaiser criterion of eigenvalue > 1, which has been described as “not psychometrically justifiable” (Reise et al., 2000, p. 291) and is prone to over and under extraction of factors (Reise et al., 2000; Russell, 2002). With parallel analysis, random data sets are generated constituting the same number of items and participants; the scree plot from these data is laid over the scree plot from the actual study data, and where the actual and simulated data lines cross indicates the *maximum* number of factors (see Figure 2); fit statistics are also taken into consideration (see Table 2). Examination of the scree plot suggested a maximum of five factors, which explained 54% of the variance. However, a four-factor solution had an adequate fit by the preferred measure (RMSEA of 0.079) and explained 50% of the variance; it was selected because the five-factor solution had ultra-Heywood cases (Dillon et al., 1987). All factor loadings for the four-factor solution are shown in Table 3. The cut-off value for a variable’s loading on a factor was set to >0.35 because this resulted in the best-fitting model and minimized the number of crossloadings.

**TABLE 2:** Goodness of fit measures and variance explained for analyses 1, 2, and 3.

	% Variance explained	RMSEA [CI]*	AGFI	CFI	TLI
Analysis 1: full sample					
EFA-4 factors	50	0.079 [0.073–0.085]			0.884
EFA-5 factors	54	0.069 [0.062–0.076]			0.914
CFA		0.081 [0.073–0.088]	0.999	0.91	0.894
SEM with predictors		0.093 [0.090–0.096]		0.75	0.694
Analysis 2: private SEM		0.093 [0.086-0.099]		0.75	0.70
Analysis 3: Montessori duration SEM		0.090 [0.085–0.094]		0.73	0.67

*90% Confidence Interval for EFA; 95% for CFA and SEM.

**TABLE 3:** Promax rotated factor loadings: EFA 4-factor solution.

Factors	Item	Factor loading				*h* ^2^
		1	2	3	4	
**Factor 1: “General Wellbeing”**						

	Satisfaction with life	**0.85**	0.01	–0.07	–0.15	0.68
	PWB_Self acceptance	**0.71**	0.16	0.03	0.01	0.72
	PWB_Environmental mastery	**0.63**	–0.04	0.05	0.23	0.61
	Subjective vitality	**0.51**	0.17	0.06	0.08	0.51
	Meaning in life	**0.47**	0.33	–0.14	0.04	0.47
	Mindful attention awareness	**0.38**	–0.07	–0.05	0.32	0.30

**Factor 2: engagement**						

	SWB_Social contribution	0.03	**0.65**	0.03	0.14	0.58
	PWB_Personal growth	–0.05	**0.60**	0.03	0.21	0.49
	SWB_Social integration	0.20	**0.59**	0.14	–0.20	0.55
	PWB_Purpose in life	0.12	**0.49**	–0.07	0.13	0.38
	PWB_Positive relations	0.35	**0.43**	0.02	–0.10	0.44

**Factor 3: social trust**						

	SWB_Social actualization	0.02	–0.05	**0.83**	0.02	0.68
	SWB_Social acceptance	0.02	0.24	**0.50**	–0.07	0.41

**Factor 4: self-confidence**						

	PWB_Autonomy	–0.08	0.03	–0.15	**0.55**	0.34
	Need for cognition	–0.23	0.30	0.05	**0.52**	0.37
	SWB_Social coherence	0.10	–0.12	–0.30	**0.47**	0.38

*The extraction method was maximum likelihood with an oblique (Promax with Kaiser normalization) rotation. Factor loadings above 0.35 are in bold.*

Factor 3 has only two indicators; three or more can improve stability (Thurstone, 1947), but this recommendation is more flexible for SEM models, and Kenny (2012) advises that a two-indicator factor is acceptable if the errors are uncorrelated and their loadings are set to equal each other. Therefore, in the CFA, the loadings of the two indicators were set to equal each other, and these conditions were met.

The factor correlations and variance accounted for are shown in Table 4. General wellbeing accounted for the largest proportion of variance (38%), and was strongly correlated with all three other factors, as might be expected of General Wellbeing. Engagement accounted for 30% of the variance, and was also strongly related to Social Trust and Self-confidence (*r*s or 0.52 and 0.48, respectively). Social Trust and Self confidence each accounted for 16% of the variance; their relation to each other was 0.28.

**TABLE 4:** EFA: factor correlations and proportion of variance accounted for.

	Proportion of variance	General wellbeing	Engagement	Social trust
General wellbeing	0.38	1.0		
Engagement	0.30	0.66	1.0	
Social trust	0.16	0.47	0.52	1.0
Self-confidence	0.16	0.53	0.48	0.28

**FIGURE 3:**
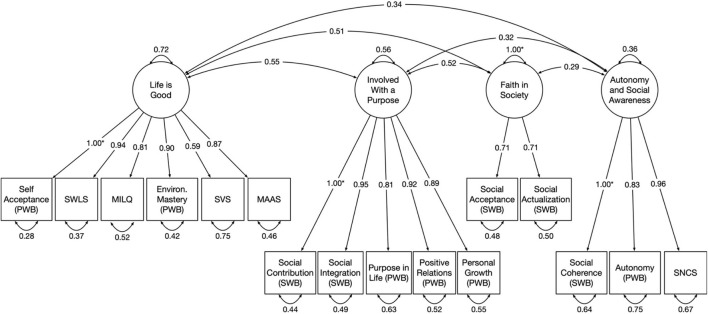
Diagram of confirmatory factor analytic solution.

Next a confirmatory factor analysis using maximum likelihood estimation was conducted on the remaining 36% of respondents’ data; the results are in Table 5 and fit indices are in Table 2. Figure 3 shows the resulting model. As one might expect from the high correlations, there were mostly very high loadings; the four factors and the item loadings are discussed next.

**TABLE 5:** CFA loadings and standard errors (SE).

Factor	Item	Estimate				
		1	2	3	4	*SE*
Factor 1: general wellbeing	PWB_Self acceptance	**1.00**				
	Satisfaction with life	**0.97**				0.04
	Meaning in life	**0.86**				0.04
	PWB_Environmental mastery	**0.90**				0.04
	Subjective vitality	**0.88**				0.04
	Mindful attention awareness	**0.62**				0.05
Factor 2: engagement	SWB_Social contribution		**1.00**			
	PWB_Personal growth		**0.88**			0.05
	SWB_Social integration		**0.91**			0.05
	PWB_Purpose in life		**0.82**			0.05
	PWB_Positive relations		**0.93**			0.05
Factor 3: Social Trust	SWB_Social acceptance			**0.71**		0.03
	SWB_Social actualization			**0.71**		0.03
Factor 4: Self-Confidence	SWB_Social coherence				**1.00**	
	PWB_Autonomy				**0.86**	0.09
	Need for cognition				**0.99**	0.10

*Factor loadings are in bold.*

### The Four Factors

The purpose of the factor analyses was to reduce the set of scales to a smaller number of factors and to suggest latent variables emerging from a set of wellbeing measures administered to all participants. Based on prior research concerning the outcomes or at least associates of self-determination, meaningful activities, and social stability (which can exist to varying degrees in any school experience), we hypothesized three latent clusters would emerge: one concerning happiness, meaning, and self-confidence; one concerning engagement and seeking challenge; and a third concerning social trust and sense of community. The factor analysis actually resulted in four factors that align reasonably well with what was hypothesized, with the first cluster of outcomes splitting into two (self-confidence and general wellbeing; see Figure 1). Again, the present study is not directly testing the paths shown in the figure; it only tests their plausibility.

#### General Wellbeing

The first factor is “General Wellbeing,” with six indicators (see Table 5). Self Acceptance from the Psychological Wellbeing Scale was set to 1.0, and one other Psychological Wellbeing scale, Environmental Mastery (e.g., “In general, I feel I am in charge of the situation in which I live”), also loaded highly on this factor (0.90). Meaning in Life (“My life has a clear sense of purpose”) and Satisfaction with Life (“In most ways, my life is close to ideal”) loaded highly on this factor as well, as did Subjective Vitality (“I have energy and spirit”). Mindful Attention Awareness (“I rush through activities without being really attentive to them” [item is reverse scored]) had the lowest loading at 0.62. Cronbach’s alpha for these six items on the CFA test data set was 0.88 (95% confidence interval 0.87–0.89). Dropping any item except Mindful Attention Awareness resulted in a lower alpha; dropping that item slightly increased alpha to 0.89, but this was within the confidence interval, and dropping it from the model resulted in Heywood cases, so it was retained.

We had hypothesized that three Montessori features–self-determination, meaningful activities, and social stability–would lead to happiness, a sense of meaning, and self-confidence. These predictions were upheld, in that the Satisfaction with Life scale, Subjective Vitality, and Self Acceptance all reflect happiness, Meaning in Life is eponymous, and Environmental Mastery reflects self-confidence. However, three other variables that reflect self-confidence in a somewhat different sense loaded instead on a discrete factor we called Self-Confidence (discussed below); in the EFA Environmental Mastery also loaded on that factor (0.23) but not at a level that met the threshold of 0.35.

#### Engagement

The second factor reflects investing oneself in one’s activities and social world. Social Contribution (“My daily activities contribute to something worthwhile to my community”) was set to 1.0 and Social Integration (“I feel close to other people in my community”) had a loading of 0.91; both these scales are from the Social Wellbeing scale. Also loading on Engagement were Personal Growth (e.g., “Life is a continuous process of learning,” 0.88), Positive Relations (“People would describe me as a giving person, willing to share my time with others,” 0.93), and Purpose in Life (“Some people wander aimlessly through life, but I am not one of them,” 0.82), all Psychological Wellbeing scales. Cronbach’s alpha for these five items was 0.83 (95% confidence interval 0.81–0.85). Dropping any item resulted in a lower alpha. This factor resembled the second cluster of outcomes we hypothesized would result from the Montessori characteristics of self-determination and meaningful activities, with a stronger social engagement element than was anticipated.

#### Social Trust

The third factor included two Social Wellbeing subscales, Social Acceptance and Social Actualization, which reflect trust in society—items like, “The world is becoming a better place for everyone” and “I believe that people are kind.” Because there were two items for this factor, their loadings were set to be equal (see above) at 0.71. Cronbach’s alpha for these two items was 0.67 (95% confidence interval 0.63–0.72). This factor reflects outcomes prior research suggested would result from Montessori’s high degree of social stability.

#### Self-Confidence

Loading on the fourth factor were three variables that reflect confidence in one’s own thinking (as opposed to one’s behaviors, which the Environmental Mastery subscale taps more). Social Coherence from the Social Wellbeing scale, including items like “I find it easy to predict what will happen next” was set to 1.0, and the Need for Cognition scale loaded highly with it (0.99); this scale includes items like, “I like to have the responsibility of handling a situation that requires a lot of thinking” and “I prefer my life to be full of puzzles that I must solve.” Autonomy from the Psychological Wellbeing scale also loaded on this factor (0.86), with items like, “I have confidence in my own opinions” and “I judge myself by what I think is important, not what others think is important.” Cronbach’s alpha for these three items was 0.57 (95% confidence interval 0.52–0.63). Dropping any item resulted in a lower alpha.

In sum, two of the three outcome clusters we had hypothesized, based on prior research, would stem from experiences involving high levels of self-determination, meaningful activities, and social stability were upheld, with Engagement having a social aspect that was not expected. The first hypothesized factor, however, split into two, with outcomes pertinent to general wellbeing (including confidence in one’s abilities) falling into one cluster and outcomes more specifically related to self-confidence about one’s thought processes in a fourth cluster.

### Predicting the Structural Model From Montessori Attendance

Having reduced the wellbeing scales to a set of four latent factors, the next step was to examine whether experience with Montessori schooling is associated with participants’ scores on any of those four factors, for the whole sample of 1905 participants, divided into the Montessori and Conventional school groups as explained above. Gender (Male/Female), race (Caucasian/Not), age, childhood SES (lower/working, lower middle, middle, upper middle, upper), and proportion of schooling that was private were accounted for as covariates in the models. There was a significant improvement in model fit when the binary variable of schooling (Montessori for at least 2 years, or Conventional) was added into the model, both overall and ranging across all four factors. Social trust had the largest beta-value (0.32) but even the lowest beta-value, for Self-confidence, showed a highly significant effect of Montessori (*p* < 0.001).

This indicates that the means on all four factors are significantly higher for the Montessori group, after accounting for the covariates. The means, SDs, standardized regression coefficients, and corrected *p*-values are shown in Table 6, and the covariate values are shown in the Supplementary Table.

**TABLE 6:** Means and SDs, standardized regression coefficients, and *p*-values for SEM.

	Mean (*SD*) Montessori	Mean (*SD*) conventional	β	*p*-value
General wellbeing	0.167 (0.83)	–0.130 (0.92)	0.17	<0.001
Engagement	0.025 (0.07)	0.019 (0.09)	0.21	<0.001
Social trust	0.370 (1.10)	–0.287 (1.23)	0.32	<0.001
Self-confidence	0.101 (0.75)	–0.078 (0.80)	0.10	<0.001

### Summary

Montessori attendance significantly predicted higher scores on all four latent variables: General Wellbeing, Engagement, Social Trust, and Self-confidence. This makes theoretical sense, in that Montessori schools have features that are related to these aspects of wellbeing. For example, Montessori gives children free choice and thus a high degree of self-determination, which (as reviewed in the section “Introduction”) has been shown in other research to render happiness and a strong sense of one’s own competence, and which allows one to find and engage in activities that give one a sense of purpose. The second feature we highlighted is that Montessori activities are meaningful, in that they have a clear purpose to which children can relate; this, along with self-determination, allows one to choose work that provides an optimal level of challenge, creating strong engagement. We did not anticipate that higher engagement among people who attended Montessori would include social integration, but it clustered with other variables tapping into engagement in the factor analysis. The third Montessori feature, social stability (including multi-year classrooms), was hypothesized to lead to strong relationships which then predict higher general wellbeing as well as social trust. Classroom looping practices also improve academic performance, which in turn predicts higher wellbeing. Thus, the results are consistent with what we hypothesized, based on prior research.

However, an alternative possible explanation for these results is that they stem not from at least 2 (and in this sample, an average of 8) years of Montessori education or some associate thereof, but instead from a third variable, perhaps something associated with having parents who make the effort to select and in most cases finance a specific school for their child, as opposed to using the default neighborhood public option. In other words, it may be that having parents who go out of their way to find and fund a different school program leads to higher adult wellbeing, or is associated with other factors that lead to higher wellbeing. Of course, many public school parents also are very intentional about their choice, choosing their domicile (and paying property taxes) based on public school district, but nonetheless they do not pay tuition in addition to taxes. Although we had covaried years of private school in the initial analysis, a more focused way to examine whether something associated with parents choosing a private school explains the results is to limit the dataset to participants who always attended a private school, because private schools are never the default option; every child in the United States and Canada lives in a school district where they could attend a tuition-free public school at least from Kindergarten on. A second analysis therefore analyzed data from the subset of participants who attended private schools for all of their schooling.

### Robustness Check/Alternative Specification #1

The second analysis involved the subsample of 439 participants who had exclusively attended private schools: a Montessori group of *n* = 268 for whom at least 2 of those years were in private Montessori (with all or most of the remaining years in conventional private school programs), and Conventional group of *n* = 171 who went exclusively to conventional private schools. The Montessori group had spent *M* = 9.22 years (*SD* = 3.59) in Montessori schools and *M* = 6.14 (*SD* = 3.71) in conventional private schools, whereas the exclusively private conventional group had spent *M* = 14.53 years (*SD* = 1.30) in school, virtually all of it in conventional private schools. The mean age of participants was 33.99 years (*SD* = 11.55, range = 18–71), 93 were male (21.2%), and the rest were female; 87.9% identified as White.

The structural equation model of the initial analysis was conducted using only data from the exclusively privately schooled subset of participants. These results, including mean factor scores and SDs, are shown in Table 7; model fit statistics are in Table 2. Even among the exclusively privately schooled subset—those participants whose parents selected and typically paid tuition at a private school for their entire precollege life, and even after accounting for the effects of age, gender, race, and childhood SES, having attended Montessori for at least 2 years (and an average of 9 years) was significantly associated with higher wellbeing on three of the four factors: General Wellbeing, Engagement, and Social Trust. Self-confidence was not significant (*p* = 0.07), suggesting that confidence in one’s own thinking/mind is as strong among those who attended private conventional schools as among those who attended private Montessori schools.

**TABLE 7:** Analysis 2, private-only sample: means, SDs, standardized regression coefficients and *p*-values for SEM.

	Mean (*SD*) Montessori	Mean (*SD*) conventional	β	*p*-value
General wellbeing	0.228 (0.77)	–0.071 (0.95)	0.17	<0.001
Engagement	0.032 (0.07)	–0.046 (0.09)	0.20	<0.001
Social trust	0.464 (1.06)	–0.184 (1.09)	0.37	<0.001
Self-confidence	0.105 (0.72)	–0.034 (0.77)	0.07	0.07

The standardized Beta values in the SEM were similar to what they were for the whole sample, but slightly stronger for Social Trust and slightly less strong for Self-confidence. Thus, while the initial analysis controlled for the proportion of schooling that was private, this second analysis shows that even among the subsample who only attended private schools, model fit improves when Montessori status is added.

Although wellbeing was still higher for Montessori compared to other participants, it is possible that this is because there is something about parents who choose *Montessori* (public or private) for their children that differs from other parents, and that it is those differences that lead to the better outcomes. This possibility was addressed in a third analysis by examining duration effects.

Particularly as children get older, duration of Montessori attendance would often reflect availability rather than parent choice. The option to attend Montessori after age 6 is limited, because Montessori elementary, middle, and high schools are far less prevalent than Montessori preschools; even Montessori elementary schools (for children ages 6–12) were extremely rare 30 years ago (when our average participant age was 6); Montessori elementary schools have gradually become more common, whereas Montessori high schools are still rare today. Because duration of Montessori attendance is often constrained by availability, self-selection is less of an issue in such an analysis, raising the odds that (were any significant effects found) the programs caused effects. Although this analysis was conducted because positive results would strengthen the possibility of causality, we caution that it is only a test of association.

### Robustness Check/Alternative Specification #2

The subset of 853 respondents from the sample who had attended Montessori for at least 1 year in childhood was examined, omitting those who had never attended it (since such an analysis would in effect virtually repeat the initial analyses). This subset had an average age of 32.07 (*SD* = 9.73, range 18–61 years). They had attended Montessori for a mean of 7.88 years (*SD* = 3.77, range = 2–16 years) and conventional school for a mean of 7.36 years (*SD* = 3.83); 23% were male and 84% were White.

The SEM tested the association between years in Montessori and the latent factors, again controlling for age, gender, race, childhood SES, and proportion of schooling that was private. The model fit statistics are in Table 2 and the regression coefficients and *p*-values are in Table 8. In this analysis, the SEM showed the duration of Montessori was significant for two of the four factors (General Wellbeing and Engagement). For all four factors, the direction was positive: being in Montessori school for longer was associated with at least slightly higher scores on all factors.

**TABLE 8:** Analysis 3, effect of Montessori duration: means, SDs, standardized regression coefficients and *p*-values for SEM.

	β	*p*-value
General wellbeing	0.10	0.01
Engagement	0.06	0.04
Social trust	0.05	NS
Self-confidence	0.04	NS

This analysis suggests two possibilities. The first is the causal possibility, that Montessori schooling could cause positive wellbeing outcomes, but that for two of the factors, a threshold number of years delivers those outcomes, and extending beyond those adds little additional benefit to social trust or self-confidence. However, General Wellbeing, composed of variables aimed at life happiness, meaning, and sense of one’s own competence, and Engagement (in one’s activities and social world) might be strengthened by more time in a Montessori environment. Alternatively, it may be that parents who choose to and are able to (because they live in communities where it is available) keep their children in Montessori longer also give their children other experiences that promote even more General Wellbeing and Engagement, but not more experiences that promote more Social Trust and Self-confidence than were indexed by the initial choice.

## Discussion

The present research aimed to determine if Montessori schooling in childhood might *plausibly* lead to higher wellbeing in adulthood, because many features of Montessori schooling are known to cause higher wellbeing contemporaneously or predictively in other school and non-school situations. Based on this existing research, we developed a hypothesized model of how three Montessori program features—self-determination, meaningful activities, and social stability—might lead to three clusters of wellbeing outcomes—a general cluster including happiness, finding meaning in life, and feeling competent; another including engaging and seeking challenge in one’s activities; and a third around a strong sense of community and social trust. Although we could not test that model directly, we could test its plausibility by seeing whether Montessori schooling was associated with latent factors aligned with those outcomes. Over 1900 individuals filled out a large set of wellbeing surveys and their responses were subjected to exploratory and confirmatory factor analyses, which arranged into four latent factors similar to those originally hypothesized, but with confidence in one’s own thoughts and mind emerging as a distinct fourth factor, and the engagement factor including social engagement as well (see Figure 1).

To test the plausibility of the hypothesis about schooling, we conducted a structural equation model analysis to determine if at least 2 years of Montessori schooling in childhood is significantly associated with adult wellbeing; the model accounted for the covariates of gender, race, age, childhood SES, and years in private school. The first analysis showed that Montessori was associated with higher scores on all four latent factors: General Wellbeing, Engagement, Social Trust, and Self-confidence.

This could be due to some feature of the parents; although the initial analysis controlled for SES, a different and unmeasured variable could be operating. The second analysis asked if something associated with selecting a private school for one’s child might be the operational variable, and therefore tested whether Montessori would be significantly associated with higher adult wellbeing even among the subsample who attended private schools at least through age 17. For three of the four latent factors, it was: General Wellbeing, Engagement, and Social Trust; the fourth, Self-confidence (in one’s thinking), showed a trend. Attending Montessori schooling for at least two childhood years was associated with higher wellbeing on these factors even among people who only attended private schools their entire pre-college lives.

Having parents who always had selected a private school was thus not responsible for the generally higher wellbeing associated with having attended Montessori observed in the initial analysis. Perhaps there is something associated with selecting Montessori specifically (whether public or private) that is associated with higher wellbeing. Although three studies that used dozens of measures (like parenting styles measures) to discriminate Montessori from other parents found no significant differences (Fleege et al., 1967; Dreyer and Rigler, 1969; Denervaud et al., 2020b), there must be some different qualities, and the present study rendered no way to examine those directly. However, Montessori enrollment, particularly as one gets older, is constrained by availability, yet the postulated unmeasured parent qualities would be expected to persist regardless of that availability. Therefore the third analysis examined whether duration of Montessori enrollment is associated with higher wellbeing. Duration of Montessori enrollment was associated with the latent factors of General Wellbeing and Engagement, but not Social Trust or Self-confidence, for which the direction of association was positive but non-significant. Either parents who choose Montessori schooling for their children, or something associated with such parents, also engenders these aspects of wellbeing, or very little (Montessori or postulated associate) exposure is needed to engender them.

However, General Wellbeing and Engagement were hypothesized to be influenced by Montessori features (see Figure 1), and are significantly and positively associated with duration of Montessori attendance (from 1 to 16 years). The latent variable of General Wellbeing was measured by scales concerning meaning in life and satisfaction with life, self-acceptance, vitality, and environmental mastery. Engagement was measured by variables tapping social contribution, social integration, positive relations, aspiring for personal growth, and a sense of purpose in life.

Although the associations with General Wellbeing and Engagement held across all three analyses, the study design does not allow one to determine if Montessori schooling caused higher wellbeing. An experimental design, in which children were randomly assigned to Montessori and then tested as adults, would be needed.

### Lottery-Control Studies

Although we know of no long-term lottery control studies of Montessori education, two short term ones were described in the section “Introduction.” The two natural experiments conducted in the US show that high-fidelity public Montessori causes features associated with higher wellbeing, like stronger mastery orientation, executive function, social knowledge/skills, and academic performance (Lillard and Else-Quest, 2006; Moffitt et al., 2011; Reynolds et al., 2011, 2017; Sancassiani et al., 2015; Steinmayr et al., 2016, 2018; Haimovitz and Dweck, 2017; Lillard et al., 2017; Darling-Hammond et al., 2019). It is also plausible that having one’s children attend Montessori changes parents, and that the parents’ subsequent behavior led to higher adult wellbeing, but these natural experiment studies lend plausibility to the hypothesized model.

### Predictive Features of Montessori for Improved Outcomes

Another support for the plausibility of the hypothesized model is that several of Montessori programs’ features, including the three highlighted here, predict higher wellbeing even when implemented in conventional school settings; these were discussed in the section “Introduction.” For example in classrooms where students are given more agency and opportunities for self-determination, they also have higher sense of their own competence and overall wellbeing (Ryan and Grolnick, 1986), and this is causal: When teachers were trained to increase students’ sense of self-determination, the students’ wellbeing increased (De Charms, 1976). Montessori work is highly engaging (Rathunde and Csikszentmihalyi, 2005a), and higher engagement leads to higher wellbeing (Csikszentmihalyi, 1990). Montessori also proffers social stability (3-year age groupings and teacher consistency) and (as indicated in research) stronger relationships (Rathunde and Csikszentmihalyi, 2005b; Lillard and Else-Quest, 2006). Strong relationships in childhood also predict higher wellbeing in adulthood (Olsson et al., 2013).

## Conclusion and Limitations

In sum, although this study only shows an association between Montessori schooling in childhood and higher adult wellbeing, lottery control studies and studies showing that features of Montessori schooling are associated with higher wellbeing in other settings lend weight to the possibility that Montessori might cause higher adult wellbeing. But if this is not the case—if in fact features of Montessori parents or some other third variable associated with Montessori attendance is the cause—then it would be very interesting to determine what the underlying cause for the discovered association is.

This study has several limitations in addition to its being a study of association rather than an experiment. One is that the sample was largely female and White, which is often the case for internet survey samples (Smith, 2008; Peytchev, 2011; Boulianne, 2013). Other studies that have tested for gender differences in Montessori outcomes typically have not found them (Lillard and Else-Quest, 2006; Culclasure et al., 2018), and children of color particularly thrive in Montessori schools (Ansari and Winsler, 2014, 2020; Brown and Steele, 2015; Brown and Lewis, 2017; Culclasure et al., 2018; Lillard et al., in press; Snyder et al., 2021). Ansari and Winsler (2014, 2020) found that only Hispanic children thrived, but many other studies show Black children thrive in Montessori as well. Furthermore, race and gender were accounted for in our models. Still, in an ideal sample, gender and race would be representative of the population, and future research should strive for a representative sample.

A second limitation concerns variation in Montessori implementation. Although the core Montessori features we discussed—self-determination, meaningful activities, and social stability—likely characterize all Montessori schools, variations in implementation might accentuate or mitigate them. For example, we have seen Montessori elementary classrooms that require students to fill out checklists of their work activities in ways that likely reduce feelings of self-determination in those classrooms. Studies showing the strongest Montessori outcomes involve high fidelity Montessori implementation (Lillard, 2019). Here, we have no information regarding the fidelity of implementation in the classrooms the adults attended. In future research, it would be useful to gather information on implementation fidelity and examine whether it varies with student wellbeing.

Another limitation is that participants knew the purpose was to consider the impact of alternative schooling on one’s life, and this could have biased people’s responding, although the direction such bias might take is unclear. If one has fond memories of school, whether it was conventional or Montessori, and one knows one is doing a survey about the impact of school on current wellbeing, one might answer more positively; if one has negative memories, one might answer more negatively. Thus, while it is unclear the direction in which knowledge about the survey’s purpose might bias any individual’s responses, in the future one might administer surveys without providing a description of the purpose. This might be difficult to do while obtaining a large sample of alternatively schooled individuals, since alternative schooling is relatively rare (based on other data from our laboratory, we estimate that 5% of American college students attended a non-conventional school at some point). Participants did not answer questions about the types of schools they attended and when until the end of the survey, which might have reduced attention to this aspect of the study until the surveys were complete, but biased responding is a limitation in survey research, particularly when it concerns subjective qualities.

Yet another limitation comes from recruitment itself. In order to get a large enough Montessori sample, Facebook ads were run in communities where Montessori schools are more abundant, such as Washington D.C., Minneapolis/St. Paul, and Milwaukee which have long had Montessori teacher training programs. Although these ads should also have recruited conventionally schooled people in those cities, it is conceivable that the study’s external validity is compromised by the strategy, because there are regional differences in wellbeing (Lawless and Lucas, 2011). For example, at the county level, life satisfaction is highly positively correlated with household income, and negatively correlated with the percentages of persons living in poverty and unemployed. The cities where Facebook ads were run varied in different ways on these metrics. For example, relative to the US average in 2018, there were higher rates of poverty and unemployment and lower median income in Milwaukee, favorable levels of all three metrics in Minneapolis, and varied levels in DC (high poverty coupled with low unemployment and high median income). Such regional variation could in part explain the levels of wellbeing in the adults sampled here, and future research should control for region to measure its contribution to wellbeing.

A further limitation concerns internal validity: We asked people to recall what type of school they attended each year from when they were 2–17, with seven options ranging from Did Not Attend to Homeschool. Some people might not have remembered their school type but guessed a type, which would produce noise in the data, rendering our results less reliable. In general, people’s memories for childhood experiences are thought to be “substantially accurate” (Brewin et al., 1993, p. 84), and memories for what type of school one attended, for almost a full year, during each childhood year, is likely to have been rehearsed in the family, lending a degree of confidence to the generally accuracy of the school data. Still, there are likely to be some inaccuracies in the data regarding school history.

In sum, wellbeing is a multidetermined but very important human outcome. If childhood schooling were to influence adult wellbeing, the public health implications would be very important. Pedagogical environments that support children to become adults with high levels of wellbeing are desirable. Although there are important limitations, the research presented here suggests that Montessori schooling might be associated with higher adult wellbeing, and that a causal relation between Montessori schooling in childhood and wellbeing in adulthood is at least plausible.

## Data Availability Statement

The raw data supporting the conclusions of this article will be made available by the authors, without undue reservation.

## Ethics Statement

The studies involving human participants were reviewed and approved by the University of Virginia Institutional Review Board for the Social and Behavioral Sciences. The patients/participants provided their written informed consent to participate in this study.

## Author Contributions

AL conceived of the study, contributed theory, raised funds, oversaw the entire project, did some data analysis, and wrote the manuscript. MM conducted the data analyses. DV and EF selected stimuli in collaboration with AL, recruited participants, put the surveys on Qualtrics, ran the study, worked with MM to prepare the data file, and contributed to the manuscript. DV ran some analyses. All the authors contributed to the article and approved the submitted version.

## Conflict of Interest

The authors declare that the research was conducted in the absence of any commercial or financial relationships that could be construed as a potential conflict of interest.

## Publisher’s Note

All claims expressed in this article are solely those of the authors and do not necessarily represent those of their affiliated organizations, or those of the publisher, the editors and the reviewers. Any product that may be evaluated in this article, or claim that may be made by its manufacturer, is not guaranteed or endorsed by the publisher.
