# Supplementary material for: An Association Between Montessori Education in Childhood and Adult Wellbeing
Source: Front Psychol. 2021 Nov 25;12:721943. doi: 10.3389/fpsyg.2021.721943 (PMC8656358; doi:10.3389/fpsyg.2021.721943)
Supplement: Supplementary file 1 [file Data_Sheet_1.pdf]

Supplementary Table. Study 1 Covariate Values in Regression for Structural Equation Model.

**DEPENDENT VARIABLE**		**Estimate**	**Std.Error**
General Wellbeing	Montessori_vs_Conventional	0.17415935	0.02276473
Engagement	Montessori_vs_Conventional	0.20530364	0.02015301
Social Trust	Montessori_vs_Conventional	0.31596907	0.03432915
Self-Confidence	Montessori_vs_Conventional	0.09971467	0.02059133
General Wellbeing	Age	0.18192111	0.02145541
Engagement	Age	0.13451746	0.0189945
Social Trust	Age	0.0718576	0.0317396
Self-Confidence	Age	0.15095303	0.01950771
General Wellbeing	SES_childhood_L	0.42525893	0.07971017
Engagement	SES_childhood_L	0.45736848	0.07000929
Social Trust	SES_childhood_L	0.54441213	0.11784553
Self-Confidence	SES_childhood_L	0.19713313	0.07103986
General Wellbeing	SES_childhood_Q	0.1144252	0.06628202
Engagement	SES_childhood_Q	0.11510588	0.0580133
Social Trust	SES_childhood_Q	0.02556312	0.09737854
Self-Confidence	SES_childhood_Q	0.09214495	0.05896133
General Wellbeing	SES_childhood_C	-0.0061491	0.05330854
Engagement	SES_childhood_C	-0.0250012	0.04665007
Social Trust	SES_childhood_C	0.00193443	0.07834657
Self-Confidence	SES_childhood_C	-0.0444035	0.04730833
General Wellbeing	SES_childhood_4	0.03764114	0.03936351
Engagement	SES_childhood_4	0.03404824	0.03446169
Social Trust	SES_childhood_4	-0.0180198	0.05784876
Self-Confidence	SES_childhood_4	0.03822608	0.03503701
General Wellbeing	GenderDummy	0.1156229	0.04835075
Engagement	GenderDummy	0.32535942	0.04269719
Social Trust	GenderDummy	0.22534789	0.07158163
Self-Confidence	GenderDummy	-0.1300742	0.04346162
General Wellbeing	TotalPropPrivate	0.04845497	0.05736434
Engagement	TotalPropPrivate	0.22281661	0.05041338
Social Trust	TotalPropPrivate	0.16423662	0.0843659
Self-Confidence	TotalPropPrivate	0.14463077	0.05104414
General Wellbeing	RaceNum	0.04690729	0.02661419
Engagement	RaceNum	0.06255602	0.02333182
Social Trust	RaceNum	0.08708495	0.03911417
Self-Confidence	RaceNum	0.04634893	0.02365566
